# Spatio-temporal trends in crop damage inform recent climate-mediated expansion of a large boreal herbivore into an agro-ecosystem

**DOI:** 10.1038/s41598-017-15438-x

**Published:** 2017-11-09

**Authors:** Michel P. Laforge, Nicole L. Michel, Ryan K. Brook

**Affiliations:** 1 Department of Animal and Poultry Science, University of Saskatchewan, 51 Campus Drive, Saskatoon, SK S7N 5A8 Canada; 2 Indigenous Land Management Institute, University of Saskatchewan, 51 Campus Drive, Saskatoon, SK S7N 5A8 Canada; 3 Present Address: Department of Biology, Memorial University of Newfoundland, 232 Elizabeth Ave, St. John’s, NL A1B 3X9 Canada; 4 Present Address: National Audubon Society, 220 Montgomery Street, Suite 1000, San Francisco, CA 94104 USA

## Abstract

Large-scale climatic fluctuations have caused species range shifts. Moose (*Alces alces*) have expanded their range southward into agricultural areas previously not considered moose habitat. We found that moose expansion into agro-ecosystems is mediated by broad-scale climatic factors and access to high-quality forage (i.e., crops). We used crop damage records to quantify moose presence across the Canadian Prairies. We regressed latitude of crop damage against North Atlantic Oscillation (NAO) and crop area to test the hypotheses that NAO-mediated wetland recharge and occurrence of more nutritious crop types would result in more frequent occurrences of crop damage by moose at southerly latitudes. We examined local-scale land use by generating a habitat selection model to test our hypothesis that moose selected for areas of high crop cover in agro-ecosystems. We found that crop damage by moose occurred farther south during dry winters and in years with greater coverage of oilseeds. The results of our analyses support our hypothesis that moose movement into cropland is mediated by high-protein crops, but not by thermoregulatory habitat at the scale examined. We conclude that broad-scale climate combined with changing land-use regimes are causal factors in species’ range shifts and are important considerations when studying changing animal distributions.

## Introduction

Global climate change is causing geographic shifts in the ranges of many wildlife species^1–3^. This highlights not only the growing need to monitor shifts in abundance and spatial distributions of animal populations but also to investigate causal factors explaining such shifts. While the predominant trend is for species to respond to a warming climate by moving either polewards or towards higher elevations^4–6^, some species are seeing their ranges expand away from the poles. Moose (*Alces alces*), are widely considered to be a boreal species with a circumpolar distribution^7,8^. However, over the past ~30 years, moose have become widespread across agricultural regions in the Boreal Plains and Prairie ecozones of Western Canada, areas typically considered highly unsuitable moose habitat and where transient animals were historically observed extremely rarely^9,10^. To understand ultimate causes for the encroachment of a species into a novel habitat, it is crucial to understand proximal causes underlying shifts in local distribution over larger scales. We propose that for moose, changes in distribution may be mediated by availability of thermoregulatory habitat (ponds and wetlands) and forage availability (e.g., crops).

Large-scale fluctuations in climate are known to affect spatial distribution, abundance, and population dynamics of many species^11,12^. The North Atlantic Oscillation (NAO), which affects winter precipitation and temperatures in North America and Europe, has been shown to broadly impact many ungulate species including moose^13,14^, white-tailed deer (*Odocoileus virginianus*)^13^, caribou (*Rangifer tarandus*)^15^, and muskoxen (*Ovibos moschatus*)^15^. Such climate indices are useful for linking population dynamics across populations at broad spatial scales throughout a species’ distribution^12^, making such analyses ideally suited to the investigation of species’ range expansion such as that of moose in agro-ecosystems.

Ungulates such as white-tailed deer, mule deer (*O. hemionus*) and pronghorn antelope (*Antilocapra americana*) are well adapted to, and often associated with, human-altered landscapes, especially agricultural areas^16–18^. Ungulates benefit from edge habitat, which often provides a mix of foraging habitat (crops or grasslands) and cover habitat (forest patches)^19^. Crops provide much higher nutrition value such as high quality lipids and digestible protein than natural vegetation, and can provide a crucial nutritional supplement especially in winter when forage resources are scarce^20^. Agriculture-dominated landscapes typically have low or non-existent populations of large predators such as wolves (*Canis lupus*) and bears (*Ursus* spp.) due to habitat loss and fragmentation combined with mortalities from roads and farmers that will often kill any predator on sight^21,22^. White-tailed deer fawns have been shown to have higher survival in cropland than in forest-dominated landscapes^16^. Despite new risks to moose in agro-ecosystems such as greater hunter access and higher probability of vehicle mortality, moose appear to receive a net benefit from human-altered landscapes in agricultural areas close to the transition to the Boreal forest^23^. Despite this, moose in isolated agricultural environments remain relatively unstudied (but see Laforge *et al*.^10^), and environmental factors that explain their recent expansion into these systems remain unexplored.

Moose are a heat-sensitive species, and face heat stress at temperatures above −5 °C in winter and 14 °C in summer^24^, and as such face thermoregulatory constraints in warm, semi-arid environments like the Canadian Prairies, where temperatures often exceed 30 °C in summer. Laforge *et al*.^10^ showed that within their home ranges, moose selected for relatively small Prairie Pothole wetlands. Moose reliance on these wetlands, combined with our field observations of moose lying neck deep in the water at the centre of these wetlands on hot days (Brook unpublished data) suggests a link between broad-scale wetland recharge mediated by long-term trends in climate and moose persistence in agricultural ecosystems. This underscores the importance of not only direct, but also indirect effects of broad-scale climate on species distributions^12^. This suggests a trade-off for moose at the level of their geographic range, where suitable foraging habitat must be balanced against thermoregulatory constraints imposed by a warming planet.

Ungulates provide economic and tourism benefits from hunting, photography and wildlife-viewing, as well as simply the intrinsic value of having wildlife present. However, free-ranging ungulates cause considerable damage to crops^17,25^ and are the primary source of damage to crops in North America^26,27^. They can also transmit diseases to both livestock^28^ and people^29^, and pose a significant danger for motorists involved in collisions^30^. Damage to crops by ungulates are an important economic concern^17^, underscoring the need for monitoring spatial and temporal trends in ungulate crop damage and providing a means to do so using crop damage claims reported to and verified by provincial crop insurance agencies.

Our knowledge of broad-scale habitat selection patterns and drivers of range expansion in moose is currently limited, especially in agriculture-dominated landscapes. We predicted that moose expansion into agro-ecosystems was mediated by thermoregulatory constraints and availability of higher than naturally occurring forage (e.g., crops). We therefore predicted that, due to its influence on precipitation regimes and subsequently the effect on recharge of pothole wetlands, the NAO would be an important mediator of moose expansion into agricultural systems. We predicted that this would be reflected in landcover use, with moose crop damage occurring in landscapes with more wetland habitat. We also predicted that moose would select for landscapes with high crop cover, as these represent an important food source for moose in this system. We also predicted that patterns in crop planting in the study area would also predict latitudinal distribution of moose crop damage. Our goal was to use verified crop damage records throughout the cultivated landscape of the Canadian Prairies to test these predictions, as crops are the dominant cover type in our study area.

## Results

### Range shift analyses

**Figure 1:**
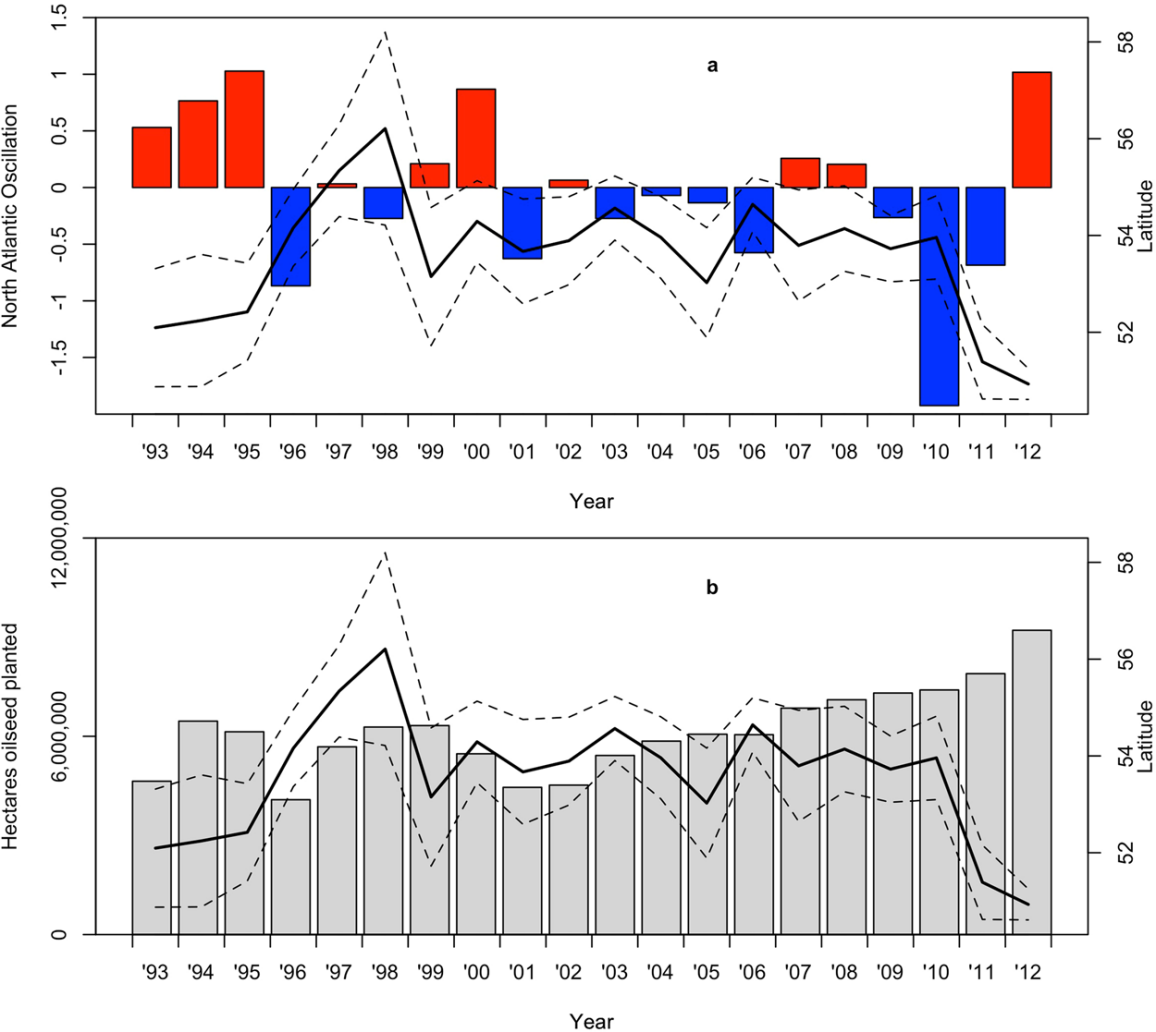
Mean latitude of crop damage by moose (*Alces alces*; *n* = 438; black line) as a function of year and associated 95% confidence interval (dashed line). Data plotted next to (**a**) North Atlantic Oscillation (blue bars are negative values representing dry winters; red are positive values indicative of wet winters) and (**b**) area of oilseeds planted in hectares across the prairie provinces of Canada (AB, SK, and MB).

We used the latitude of crop damaged by moose (expressed as the percentile-rank of latitude as a function of the latitude of all crop in the study area) as the response variable in our analyses. Crop damage was regressed against total area planted of both grain and oilseed crops as well winter-averaged North Atlantic Oscillation (NAO) data. We also evaluated the influence of NAO in winter of the two previous years. The best model of the candidate set (lowest AIC; see Table 1), was a model that included both NAO (in the current year), as well as proportion oilseeds planted. This model had a negative value for both current year NAO (β [95% CI] = −0.055 [−0.080, −0.030]) and total seeded area of oilseeds (β [95% CI] = −0.079 [−0.104, −0.054], see Table 2). This indicates that moose moved south when the current year had high (positive) NAO (cold and dry), and north with a low (negative) value (wet and warm). Moose also damaged crops further south as overall area of oilseeds planted increased. Crop damage latitude and 95% confidence intervals are plotted in Fig. 1, along with NAO phase and area of oilseeds planted. The top model (using percentile-ranked data) had better explanatory power (R^2^ = 0.132) compared to the top model using raw latitude only (R^2^ = 0.098), see Table 1 and S1.

**Table 1:** Top models, degrees of freedom (df) and ΔAIC for moose (*Alces alces*) crop damage claim (*n* = 438) percentile-ranked latitude as a function of area of oilseeds and grains planted and the North Atlantic Oscillation (NAO) quantified at the year in which damage occurred and at one- and two-year lags in the agricultural regions of AB, SK and MB.

Parameters	df	ΔAIC	Weight
Oilseeds + NAO	4	0.000	0.570
Oilseeds + NAO + NAO_t−1_	5	1.422	0.280
Oilseeds + NAO + NAO_t−1_ + NAO_t−2_	6	3.338	0.107
Grains + NAO	4	6.475	0.022
Grains + NAO + NAO_t−1_	5	7.327	0.015
Grains + NAO + NAO_t−1_ + NAO_t−2_	6	9.281	0.006
Oilseeds	3	16.583	0.000
NAO + NAO_t−1_ + NAO_t−2_	5	24.291	0.000
NAO	3	35.331	0.000
Grains	3	47.495	0.000
Intercept only	2	59.931	0.000

**Table 2:** Parameter estimates and 95% confidence interval (CI) for the top model for moose (*Alces alces*) crop damage claim (*n* = 438) latitude in the agricultural regions of AB, SK and MB, Canada for models explaining changes in crop damage latitude (expressed as the percentile of crop damage latitude as a function of all crops in the study area) as a function of the North Atlantic Oscillation (NAO) and area of oilseeds planted in the current year.

Parameter	β	95% CI		p
		Lower	Upper	
Intercept	0.719	0.694	0.743	<0.001
Oilseeds	−0.079	−0.104	−0.054	<0.001
NAO	−0.055	−0.080	−0.030	<0.001

### Habitat selection

**Figure 2:**
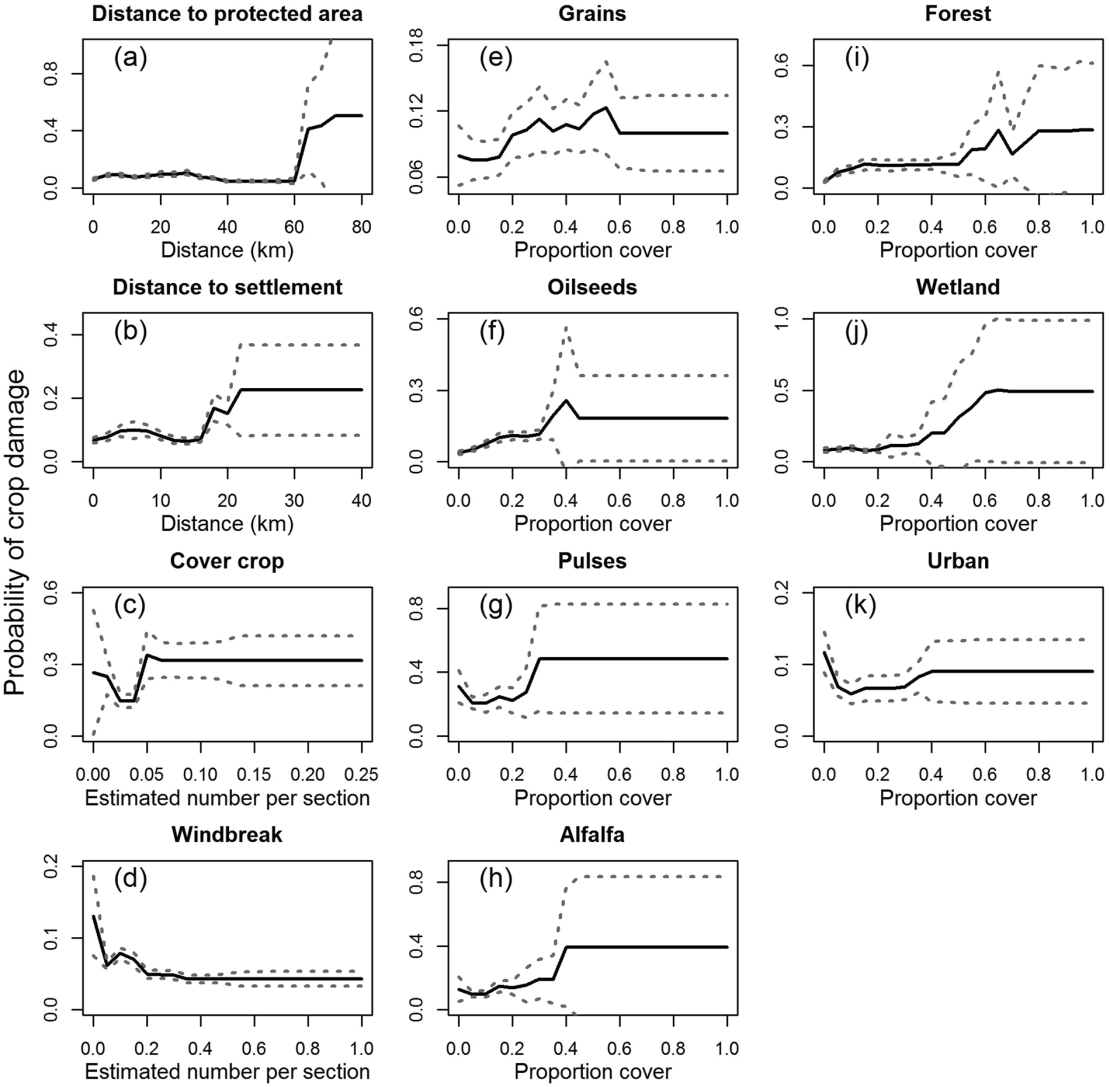
Marginal predicted probabilities of crop damage by moose (*Alces alces*) across cultivated regions of the Prairie and Boreal Plains ecozones of Canada, 1993–2012.

We used boosted regression tree (BRT) models to evaluate habitat selection patterns of moose in the study area as a function of landcover covariates. The final BRT model accounted for 59.4% of the deviance in our data, with an AUC of 0.976 and 5-fold correlation between spatially-distinct datasets of *r*_*s*_ = 0.689. The probability of moose selection of a section of land, as indicated by crop damage, was best explained by the density of cover crops and windbreaks, followed by proportion oilseeds, pulses and forests, which all explained >10% of the model deviance (Table 3). Moose exhibited a non-linear response to cover crop, initially exhibiting lower selection of sections with farms that planted cover crops followed by increased probability of selection, after which selection probability was unaffected. Moose avoided sections with a high density of farms with windbreaks. Probability of moose selection increased with cover of pulses, oilseeds and alfalfa (Fig. 2). Moose habitat selection showed a non-linear relationship with proportion grains, with greatest risk occurring at intermediate levels of grain in the section. Moose also exhibited a slight positive response to wetland and forest cover (Fig. 2).

**Table 3:** Relative contribution and overall trend of predictor variables for boosted regression tree models of moose (*Alces alces*) crop damage across cultivated regions of the Prairie and Boreal Plains ecozones of Canada, 1993–2012.

Predictor	Relative Contribution (%)	Trend
Number of farms with cover crops	14.0	+
Proportion oilseeds	12.6	+
Number of farms with windbreaks	12.4	−
Proportion pulses	11.0	+
Proportion forest	10.1	+
Distance to nearest settlement	8.7	+
Distance to nearest protected area	8.4	+
Proportion alfalfa	6.2	+
Proportion wetland	6.1	+
Proportion urban	5.3	−
Proportion grains	5.0	+

**Figure 3:**
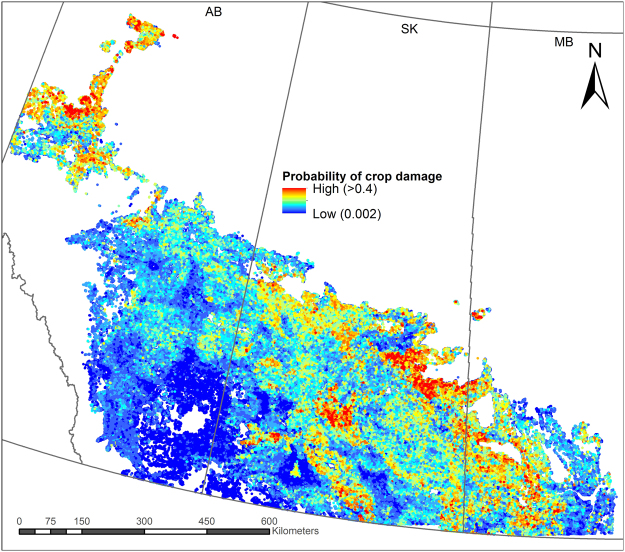
Spatial distribution of predicted crop damage by moose (*Alces alces*) across cultivated regions of the Prairie and Boreal Plains ecozones of Canada based on a resource selection function model using boosted regression tree analysis. Coloured/shaded areas have agricultural crop cover; white areas have no crop production. The map was based on landcover data from 2010 and produced using ArcMap version 10.2 (Environmental Systems Research Institute; www.esri.com).

Moose avoided human settlements, as selection probability increased with distance from the nearest town or village, and declined in response to proportion cover by urban features. Selection probability increased dramatically in areas >60 km from the nearest protected area; however, sections >60 km from protected areas were rare, representing <1% of both moose presence and absence records, and consequently the confidence intervals were quite large, indicating high model uncertainty (Fig. 2). Predicted probabilities of moose crop damage (based on landcover data from 2010) are plotted in Fig. 3.

## Discussion

Broad-scale climate indices are known to have direct effects on animal resource selection patterns. We predicted that moose range expansion into farmland habitat was mediated by thermoregulatory constraints and by forage availability. NAO was an important predictor of the latitudinal distribution of crop damage by moose in the Canadian Prairies, although the relationship was opposite to what we had predicted—moose moved south in high NAO years, suggesting that colder and drier winters were more conducive to southerly expansion of moose range. At the landscape scale moose selected for areas of high crop cover (especially winter cover crop), wetland, and forest and avoided urban areas and areas with windbreaks. Moose displayed overall similar selection patterns for most crop types; however, oilseeds and pulses—crops of high caloric value^31^—had the highest variable importance.

We found that moose crop damage occurred farther north in warm, snowy winters. This could be an indication of thermoregulatory constraints on moose, if warmer winters associated with a positive NAO phase result in conditions that are sub-optimally warm for moose, which can face heat stress in temperatures warmer than −5 °C in winter^24^. Northward movement observed in snowy winters may also suggest reduced forage abundance for moose or restricted mobility due to more significant snowpack^32^. In reindeer (*R. tarandus*), population growth rate was found to be significantly negatively correlated with annual winter precipitation^33^.

We found little support for our hypothesis that moose latitudinal shifts were mediated by overall availability of wetlands. Despite marginal support from our boosted regression tree analysis on crop damage as a function of wetland abundance (Fig. 2j), results from our regression analysis on NAO were counter to this prediction. This indicates that wetland abundance in the Canadian prairies may not be a limiting factor for moose (at least not over the timescale of this study), despite strong evidence from Laforge *et al*.^10^ who found that at a fine scale (100 m) moose in agricultural systems select for wetlands in both summer and winter. This is likely explained by the widespread distribution of Prairie Pothole wetlands across the landscape such that they are widely available throughout most of our study area and locally selected by moose at fine scales.

Moose showed intermediate selection for most cover types (Fig. 2), suggesting that moose selected for heterogeneous environments, consistent with the forage–cover trade-off hypothesis^34,35^. Forest cover and wetlands provide important cover habitat for thermoregulation and protection from predators and hunters, while crops provide abundant forage. Probability of crop damage increased nonlinearly with crop cover, suggesting that moose actively selected for crops rather than exhibiting passive increases in crop damage with crop availability. This supports our hypothesis of moose range shifts being driven by availability of forage crops.

Our model of crop damage latitude showed that moose damaged crops further south as area of oilseed crops increased. Area of oilseed crop was negatively associated with area of grain crop, necessitating use of only one in our models and also suggesting that moose damage occurred farther north as area of grain crops increased. Canola (the most abundant oilseed crop in the study area) has a much higher protein content (26%) than grains (oats: 9%, barley: 7%)^31^, and diets high in oilseeds have been shown to result in weight gain^36^ and increases in fatty acids^37^ in domestic ruminants. Oilseeds were also the most commonly used crop by moose in our study area (see Supplementary Table S2). These results support our hypothesis of moose moving into farmland areas to exploit high nutrition crops.

Winter cover crop was notably found to be an important predictor of moose habitat selection. Very few studies have examined the role of cover crop in ungulate resource selection, with the exception of one study suggesting that pronghorn antelope (*Antilocapra americana*) select for cover crop due to reduced snow cover^38^. This may indeed be a factor in moose habitat selection; however, we suspect that cover crops provide valuable winter forage for moose in the form of spilled grain, unharvested grain swaths, and perennial crops that remain in fields.

At broad spatial scales, moose appear to benefit from human-altered landscapes. In Norway, both forestry and agricultural operations have provided key foraging opportunities for moose^35^. In the Peace River region in northwestern Alberta, moose density was more than 1.5 times higher in agricultural/settled regions than in the surrounding undisturbed boreal habitat^23^. Moose avoidance of areas with a high proportion of urban/developed cover and selection for areas far away (>20 km) from the nearest settlement supports the assertion that moose selection for human-altered landscapes is hierarchical, with moose preferring them at broad spatial scales but avoiding human infrastructure at intermediate (i.e., section level) and fine scales^10^. Moose avoided areas with a high number of windbreaks. Previous studies have suggested that windbreaks may provide cervids with food and cover^39^ as well as potential fawning areas^40^; however, windbreaks may also be used by hunters^40^, or may simply be associated with higher human density and more urban areas and farmyards. Windbreaks have declined considerably in farmland areas in Western Canada in part due to shifts in agricultural practices such as no-till, which leaves crop residue on the land to prevent wind erosion, though also leaving more grain accessible to moose in winter.

In addition to providing insight into drivers of multi-annual variation in range limits in moose, our results also suggest possible drivers of overall range expansion at multi-decadal scales. Higher densities of people in rural areas in the early 20^th^ century may have discouraged moose from encroaching into farmland, despite the emergence of a new and potentially valuable resource (agricultural crops). Increased urbanization in the Canadian prairies has likely subsequently made cropland more attractive to moose, with dominant crop type and NAO-mediated winter severity mediating this expansion at multi-year scales.

Knowledge of ungulate distribution and range shifts is invaluable to managing human–wildlife conflict in agricultural regions. Crop damage by ungulates and the risk of ungulate-vehicle collisions have generated considerable wildlife–human conflict and significant economic costs^17,25,41^. Understanding factors associated with the apparent success of moose in farmland will help elucidate risks and factors associated with expanding moose populations. Conventional wisdom has dictated that increased access to moose by hunters is an important driver of population decline in forested areas, yet these farmland moose are thriving despite having by far the highest levels of hunter access compared to any boreal forest moose population.

Understanding broad-scale habitat selection patterns as well as temporal variation in species’ ranges is invaluable in continuing to understand how populations will adapt to changing climate and land-use regimes. Our results demonstrate the importance of integrating broad-scale climate indices with regional-scale land-cover to understand species’ range expansion and contraction over large geographic scales. Such knowledge is crucial to our understanding of future range shifts in a changing global climate.

## Methods

### Study area and farmland moose

**Figure 4:**
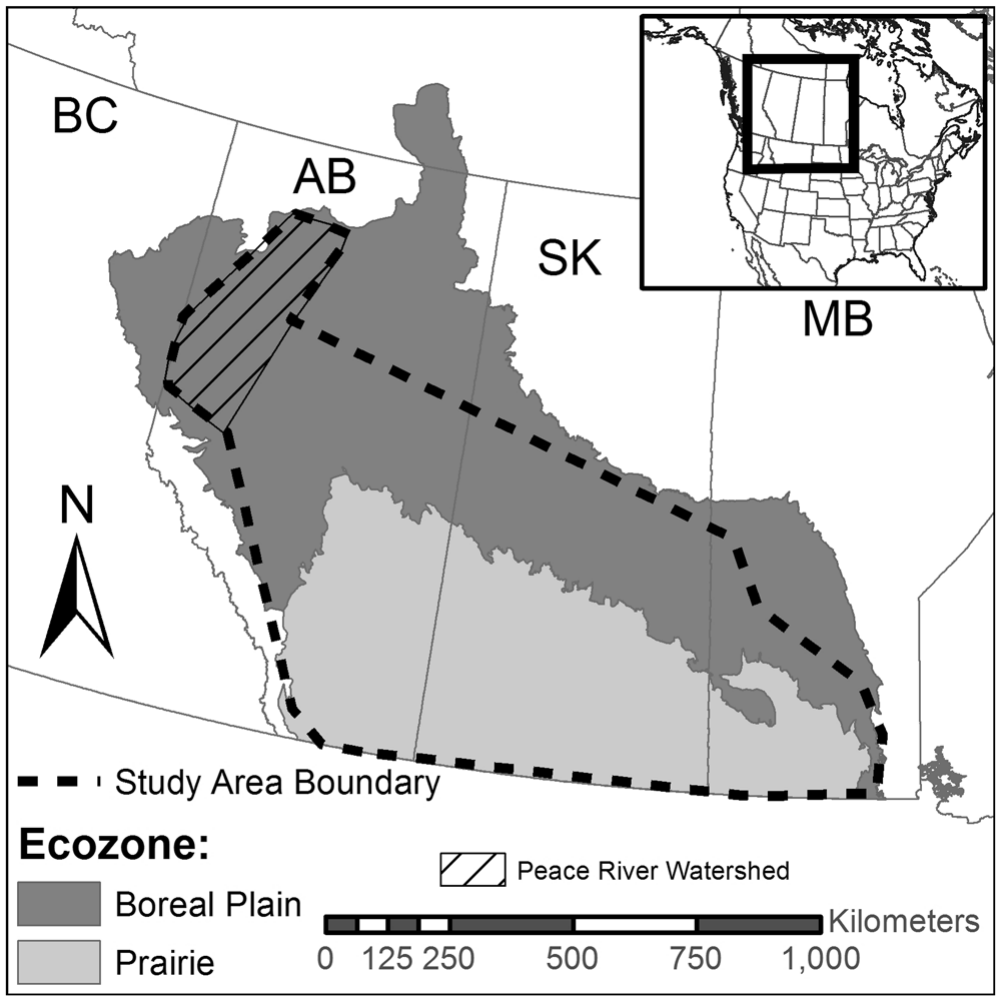
Location of study area across cultivated regions of the Prairie and Boreal Plains ecozones of Canada. The dashed line encompasses the agricultural areas for which crop damage claims by moose occurred during our study (1993–2012). The map was produced using ArcMap version 10.2 (Environmental Systems Research Institute; www.esri.com).

Our study area consisted of agricultural regions (sections of land with non-zero values for proportion of agricultural crop cover) of the Prairie and Boreal Plains ecozones across Alberta (AB), Saskatchewan (SK) and Manitoba (MB), encompassing ~800,000 km^2^ (Fig. 4). The Prairie ecozone is intensively cultivated with 68% cropland (annual and perennial) within the study area in 2010, compared to 23% cropland across the Boreal Plains region of the study area (Table 4)^40^.

**Table 4:** Percent cover of different landcover classes for the study area in both the Prairie and Boreal Plains ecozones.

Class	Prairie (%)	Boreal Plains (%)
Cropland	68.4	23.4
Grassland	17.6	0.1
Forest	5.8	62.8
Water	4.5	5.2
Wetland	1.4	7.3
Roads	1.3	0.7
Settlement	0.9	0.4

The area is populated by moose, which constitute a relatively recent arrival in this ecosystem. Data from collared moose in this system have shown significant selection for wetlands and forests^10^. These moose are non-migratory despite some changes in resource selection patterns throughout the year^10^.

### Input datasets

Given the extensive coverage of crops within the study area, we used locations of crop damage claims that were verified by trained government staff damage adjustors from each provincial agency and recorded systematically using standardized criteria and stored in databases by each of the provinces of Alberta (1993–2010; Agricultural Financial Services Corporation, www.afsc.ca), Saskatchewan (2000–2012; Saskatchewan Crop Insurance Corporation, www.saskcropinsurance.com), and Manitoba (1989–2009; Manitoba Agricultural Services Corporation, www.masc.mb.ca) as indicators of moose presence. These compensation programs are funded jointly by the Canadian federal government and each provincial government and employ field staff to verify claims prior to payment of normally 100% of the verified loss due to wildlife. Past studies have evaluated these data and have found them to correspond well with other datasets such as GPS satellite collar data^42,43^ and provide broad coverage of entire provinces over multiple years and represent the best and likely only available data for provincial- and national-scale understanding of species’ distribution on farmland across multiple years. Brook^41^ found that <1% of farmers with crop damage do not report their losses since losses are typically measured in thousands of dollars (CDN). Crop damage claims are reported at the quarter-section level (0.8 × 0.8 km, 0.64 km^2^) based on the Canadian Dominion land grid where land has been delineated and ownership is typically based on purchasing individual quarter sections. We aggregated reported crop damage claims to sections (1.6 × 1.6 km, 2.6 km^2^) as this was the highest resolution available for crop damage data from Manitoba and summed the number of reports from individual sections within each year. Our final dataset included 819 crop damage reports on 438 sections during 1993–2012. Distribution of crop damage by crop type is presented in Table S2.

We used the North Atlantic Oscillation (NAO) to examine climate-induced latitudinal shifts. The NAO typically has a greater effect in the winter months (Dec–Mar)^44^, therefore we averaged monthly NAO values across those months. The NAO influences temperature and precipitation in Europe as well as North America. Many studies have demonstrated that high, positive values are associated with warm, wet winters in North America, whereas low, negative values are associated with dry, cold winters^11,12,45^. We calculated current year NAO as well as one- and two-year lags (ftp://ftp.cpc.ncep.noaa.gov/wd52dg/data/indices/nao_index.tim). Area of oilseeds (canola and flax) and area of grains (wheat, barley and oats) planted each year were obtained from Statistics Canada (www.statcan.gc.ca).

Landcover and crop types were assessed at the section level. As the study period spanned 20 years, during which time land use may change, we used three Agriculture and Agri-Food Canada (AAFC) datasets to assess landcover. We used the 1990 dataset to assess use and availability for data years 1993–1995, the 2000 dataset for 1996–2005 and the 2010 dataset for 2006–2012^46^. We reclassified each dataset to eight landcover classes: forest, cropland, managed grassland, unmanaged grassland, wetland, water, urban, and other, and calculated the proportion cover of each class at the section level using ArcMap 10.2 (Environmental Systems Research Institute, Redlands, CA).

Specific crop type cover data were obtained from the Canadian Census of Agriculture for Census years 1991, 2001 and 2011 respectively for our three landcover datasets. We used census data spatially interpolated to the ecodistrict level, as this represented the smallest spatial scale for which data were not censored for privacy concerns^47^. We assessed four categories of agricultural crops: annual cereal grains (including wheat, oats, barley, mixed grains, rye, grain corn, buckwheat, and triticale); annual oilseeds (including canola, flaxseed, and soybeans); annual pulses (including beans, peas, lentils, and chickpeas); and perennial forage crops, including alfalfa. Proportion cover of each crop type at the section level was assessed by first calculating the proportion of all crop lands at the ecodistrict level composed of each crop type. These crop type proportions were then multiplied by the proportion cover of cropland on the section level estimated from the AAFC dataset (above) to obtain estimates of percent crop type cover at the section level. For sections that overlapped two or more ecodistricts, we calculated weighted crop type proportions based on the section area in each ecodistrict. These same methods were used to interpolate the number of farms with windbreaks and number of farms planting winter cover crops (generally alfalfa) within each section from ecodistrict-level totals.

Additionally, we included distances to the nearest protected area and nearest settlement. We defined protected areas as publicly-managed lands where hunting was prohibited, including national parks, wildlife refuges, wildlife areas, research areas, ecological reserves, and selected recreation sites, historic sites, and provincial parks. We identified public lands that functioned as hunting refuges using provincial hunting guidelines^48–50^. Based on recommendations by Barbet-Massin *et al*.^51^, we drew a random sample of 10,000 sections (out of a total of 212,766 sections) with no moose crop damage reports as our available points against which to model moose habitat use. We assumed that due to the financial incentive for farmers to report moose crop damage to provincial authorities, sections without reports represent true absences and this is consistent with findings from Brook^52^. Each absence section was assigned to an analysis year (1990, 2000, 2010) using a random subset design such that the proportions of absence data in each province and analysis year were the same as in the presence data, and this analysis year determined the landcover and census dataset associated with each section.

### Range shift analyses

We regressed latitude of section centroid damaged by moose against current year NAO, along with one- and two-year lagged NAO, as well as area of oilseed crop and grain crop planted in the study area to evaluate the influence of current and historic climate trends as well as planting regime to evaluate broad-scale moose range shifts. We evaluated two forms of the response variable, one using raw values of latitude of crops damaged by moose, and a transformed version of the variable that was percentile-ranked based on the latitudinal distribution of all crops in the study area. This formulation accounted for bias in the latitudinal distribution of crops across the study area. We compared the AIC values of each model set and the R^2^ value of the top model of each set to determine which form of the response variable to use. We screened explanatory variables for collinearity (>|0.7|)^53^. We found that area of oilseed planted and area of grain planted were negatively correlated, and therefore did not include both variables in any single model when building our candidate set of models. We generated a set of *a priori* models^54^ and ranked the models by Akaike’s information criterion (AIC).

### Habitat selection modelling

We used boosted regression trees to model moose habitat selection in response to the landcover, landscape, and agricultural variables described above^55^. Resource selection functions (RSFs) and resource selection probability functions (RSPFs) are commonly fit using binomial generalized linear models^56^. However, the linear modeling framework has numerous limitations when modeling spatial data, including collinearity, multicollinearity (i.e., the unit sum problem), and limited ability to model non-linear responses and interactions^57–59^. Machine learning techniques such as boosted regression trees (BRTs) have been used extensively for species distribution modeling over the last decade^58,60,61^; yet machine learning techniques have rarely been used to build RSFs or RSPFs, despite their many advantages, including their ability to effectively model non-linearities and interactions while remaining robust to pairwise- and multi-collinearity^55,58^.

Machine learning techniques have recently emerged as powerful new tools for ecologists. Boosted regression tree analysis is a form of machine learning which partitions data iteratively to generate a large number of regression trees, which are aggregated to create a robust predictive model^55,62^. Boosted regression trees are largely unaffected by multicollinearity, which can severely affect traditional regression methods^59^. They also provide a flexible means of modelling non-linear relationships and interactions^60^, which are increasingly being recognized as important to consider in ecological studies^58,60^.

BRT models were built using package dismo^63^ in R version 3.2.1^64^. The response variable (presence/absence of crop damage by moose at the section level), was weighted by the number of claims that occurred on that section each year. BRT models use three parameters—learning rate, bag fraction, and tree complexity—to shrink the number of terms in the final model and thus avoid overfitting. Learning rate shrinks the contribution of each tree in the boosted model, bag fraction specifies the proportion of data to be selected from the training set at each step, and tree complexity determines the number of nodes and, consequently, level of interactions between predictors. We iteratively tuned these parameters to optimize model fit while ensuring a minimum of 1,000 trees using default parameter ranges recommended by Elith *et al*.^55^: learning rate 0.0001–0.1, bag fraction 0.55–0.75, tree complexity 1–7. At each step we used 10-fold cross-validated area under the curve (AUC) and residual deviance to select the optimal parameter value. Our final model had a learning rate of 0.03, a bag fraction of 0.6 and a tree complexity of 6.

Model simplification (i.e., removal of redundant predictors that provide limited information) was conducted using command *gbm.simplify*, which uses 10-fold cross-validation to identify predictors that can be removed without affecting predictive performance^55^. Model fit was assessed using three measures: percent deviance explained, cross-validated AUC, and the mean Pearson’s correlation (*r*_*s*_) between binned crop damage probability scores (i.e., *k*-fold cross-validation)^65^. To reduce the influence of spatial autocorrelation on cross-validation we divided the study area into five spatially distinct regions along longitudinal lines, each with an equal number of crop damage records, and used these longitudinal blocks as the hold-out datasets in the five cross-validations^66^. We present variable importance scores, which are calculated based on the number of times each variable occurs in the set of trees weighted by its mean improvement of tree fit, to indicate the relative influence of each predictor. We produced marginal response plots depicting the model-estimated probability of selection, across the range of values for each predictor variable, with all other predictors held at their mean value. We calculated 95% confidence intervals by bootstrapping the model 1,000 times using resampling with replacement^67^. Model-estimated crop damage probability was plotted using ArcMap 10.2 (Environmental Systems Research Institute, Redlands, CA) using data from the 2010 landcover map.

### Data availability

The data that support the findings of this study are available from Alberta Agricultural Financial Services Corporation, Saskatchewan Crop Insurance Corporation, and Manitoba Agricultural Services Corporation; but restrictions apply to the availability of these data, which were used under license for the current study, and so are not publicly available. Data are however available from the authors upon reasonable request and with permission of Alberta Agricultural Financial Services Corporation, Saskatchewan Crop Insurance Corporation, and Manitoba Agricultural Services Corporation.

## Electronic supplementary material

Supplementary Tables S1 and S2
