# Supplementary material for: Spatio-temporal trends in crop damage inform recent climate-mediated expansion of a large boreal herbivore into an agro-ecosystem
Source: Sci Rep. 2017 Nov 9;7:15203. doi: 10.1038/s41598-017-15438-x (PMC5680171; doi:10.1038/s41598-017-15438-x)
Supplement: Supplementary file 1 — Supplementary Tables S1 and S2 [file 41598_2017_15438_MOESM1_ESM.doc]

**Table S1.** Top models, degrees of freedom (df) and ΔAIC for moose (*Alces alces*) crop damage claim (*n* = 438) latitude using raw latitude (not percentile-ranked) as a function of area of oilseeds and grains planted and the North Atlantic Oscillation (NAO) quantified at the year in which damage occurred and at one- and two-year lags in the agricultural regions of AB, SK and MB. The top model in this model set was 1909.3 AIC points higher than the top model using percentile-ranked data (see main text).

| Parameters | df | ΔAIC | Weight |
| --- | --- | --- | --- |
| Oilseeds + NAO | 4 | 0.000 | 0.334 |
| Grains + NAO | 4 | 0.323 | 0.284 |
| Grains + NAO + NAO_t–1_ | 5 | 1.826 | 0.134 |
| Oilseeds + NAO + NAO_t–1_ | 5 | 1.959 | 0.125 |
| Grains + NAO + NAO_t–1_ + NAO_t–2_ | 4 | 3.307 | 0.064 |
| Oilseeds + NAO + NAO_t–1_ + NAO_t–2_ | 7 | 3.532 | 0.057 |
| NAO + NAO_t–1_ + NAO_t–2_ | 5 | 12.055 | 0.001 |
| Oilseeds | 3 | 13.587 | 0.000 |
| NAO | 3 | 23.176 | 0.000 |
| Grains | 3 | 33.489 | 0.000 |
| Intercept only | 2 | 43.214 | 0.000 |

**Table S2.** Percentage of crop types damaged by moose (*Alces alces*) in the Canadian Prairies.

| Crop | % of total |
| --- | --- |
| Canola | 36 |
| Oats | 17 |
| Wheat | 13 |
| Flax | 9 |
| Field Peas | 7 |
| Sunflower | 7 |
| Barley | 5 |
| Canary seed | 4 |
| Buckwheat | 1 |
| Mustard | 1 |
